# Optimal starting age of endoscopic screening for esophageal cancer in China: A multicenter prospective cohort study

**DOI:** 10.1002/cam4.5727

**Published:** 2023-04-07

**Authors:** Ru Chen, Lizhou Dou, Jiachen Zhou, Guohui Song, Bianyun Li, Deli Zhao, Zhaolai Hua, Xinzheng Wang, Jun Li, Changqing Hao, Yanyan Li, Xiang Feng, Lin Li, Wenqiang Wei, Guiqi Wang

**Affiliations:** ^1^ National Cancer Center/National Clinical Research Center for Cancer/Cancer Hospital Chinese Academy of Medical Sciences and Peking Union Medical College Beijing China; ^2^ Department of Epidemiology and Biostatistics School of Public Health, Xi'an Jiaotong University Health Science Center Xi'an Shaanxi China; ^3^ Cixian Cancer Hospital/Cixian Institute for Cancer Prevention and Control Handan China; ^4^ Linzhou Cancer Hospital Anyang China; ^5^ Feicheng People's Hospital Taian China; ^6^ Cancer Institute of Yangzhong City/People's Hospital of Yangzhong City Zhenjiang China; ^7^ Yangcheng Cancer Hospital Jincheng China; ^8^ Yanting Cancer Hospital Mianyang China

**Keywords:** cohort, endoscopic screening, esophageal cancer, high risk areas, starting age

## Abstract

**Background:**

Although endoscopic screening for esophageal cancer has been performed in high‐risk areas in China for decades, there is limited and inconsistent evidence regarding the starting age for individuals participating in screening. The aim of this study is to investigate the optimal starting age of esophageal cancer screening.

**Methods:**

This study is based on a multicenter prospective cohort consisting 338,017 permanent residents aged 40–69 years in six high‐risk areas of esophageal cancer in China. The participation rate, detection rate, hazard ratios (HRs), cumulative incidence and mortality and number needed to screen (NNS) were calculated in each age group. Screening burden, benefit and risk were compared among screening strategies with different initiation ages to explore the optimal starting age for population‐based screening in high‐risk areas.

**Results:**

Individuals aged 50–69 had a higher participation rate, a higher detection rate and improved screening effectiveness than those aged 40–49. The endoscopic screening had no significant effect on reducing the incidence of esophageal cancer in individuals under 55 and mortality in individuals under 45. Increasing the starting age to 50 years reduced the screening demand and NNS by 40% and 55%, and resulted in 12% of detectable positive cases, 16% of preventable incident cases, and 14% of preventable deaths being missed.

**Conclusions:**

Postponing the starting age of endoscopic screening to 50 years might yield a more‐favorable balance between screening benefit and burden in high‐ risk areas with limited resources.

## Graphical abstract

Compared with the unscreened group, initiation of screening after 50 years was associated with a greater reduction in cumulative incidence and mortality in 10 years follow‐up compared with initiation before 50 years.

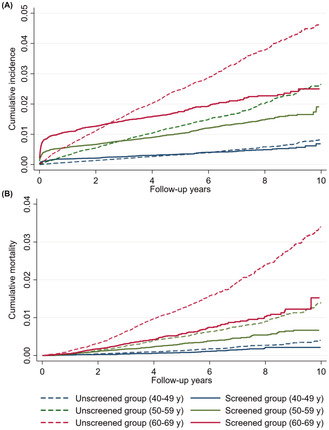

## 1 INTRODUCTION

Esophageal cancer is a global public health challenge. According to estimates from the GLOBOCAN project, there were more than 604,000 new cases and 544,000 cancer deaths worldwide in 2020.^1^ Approximately 70% of cases occur in men and more than 90% of cases are over 50 years of age.^1^ In China, although the survival of esophageal cancer has been greatly improved in the past few decades,^2^ esophageal cancer remains the sixth in terms of incidence and fourth in mortality,^3^ and continue to cause large burden on socioeconomic and human health.

Accumulating evidence has demonstrated that endoscopic examination is an effective tool for screening and can provide long‐term protection from esophageal cancer occurrence and death in high‐risk areas in China.^4^, ^5^, ^6^ The European Society of Gastrointestinal Endoscopy (ESGE) also recommended endoscopy for esophageal cancer screening in high‐risk individuals or patients (opportunistic) in the latest statement.^7^ However, endoscopic screening is limited due to high cost, low detection rate and low compliance.^8^, ^9^ Starting age is crucial to the efficiency and cost‐effectiveness of screening programs. Because of the relatively low progress from dysplasia to invasive cancer, the rapidly increase in incidence with age will lead to the increased prevalence in individuals undergoing an endoscopy screening. Meanwhile, a higher participation rate was observed in older people in previous studies,^4^, ^5^ suggesting that age may be associated with the willingness to participate in screening. Therefore, determining the appropriate starting age for screening can reduce the burden of screening and improve the effectiveness by achieving a higher detection rate and participation rate.

There is no guideline for esophageal cancer screening worldwide. The current screening program conducted in high risk areas of esophageal cancer in China recommended all permanent residents aged 40–69 years old to take at least one endoscopy.^10^ With the development in economy and improvement in healthcare services, the incidence and mortality of esophageal cancer has been decreased from 19.32/100000 and 15.39/100000 in 2005 to 17.87/100000 and 13.68/100000 in 2015 in China, respectively.^11^, ^12^ Of note, the incidence of esophageal cancer in individuals aged 40 in 2005 was about twice compared with that in 2015. Therefore, it is urgent to clarify whether the starting age in current program is appropriate, and if not, what is the optimal starting age for population‐based endoscopic screening.

Given the limited and inconsistent evidence on the optimal starting age for esophageal cancer screening,^13^, ^14^ the aim of our study was to evaluate the impact of starting age on the effectiveness of screening through comprehensive assessment on multiple outcomes, and to provide high‐quality evidence for optimization and implementation of screening programs in China and other countries with high risk of esophageal cancer.

## 2 MATERIALS AND METHODS

### 2.1 Study population

This study was based on data from a multi‐center population‐based screening cohort. The details on population selection have been described previously.^4^ In brief, the screening cohort was established in six centers located at high‐risk regions in China since 2005 and all permanent residents aged 40 to 69 years were regarded as target population. From 2005 to 2012, a total of 338,017 individuals were invited to participate in screening program, and 113,340 of them took endoscopic examination eventually.

### 2.2 Screening procedure

A once‐only endoscopic examination was offered to participants who agreed to take screening. The screening was done in local hospital by well‐trained doctors according to the screening protocol for upper‐gastrointestinal cancer in China.^15^ Endoscopy with Lugol's iodine solution was performed after local anesthetic (5 mL of 1% lidocaine by mouth for 5 min) or intravenous anesthesia. The entire esophagus was visually examined and biopsies were taken from all suspicious lesions that were unstained. Two experienced pathologists read all the biopsy slides independently without knowing the visual endoscopic findings. Any discrepant diagnoses were resolved by joint review or inviting a third pathologist to reach a consensus diagnosis. The precancerous lesions were initially diagnosed as mild dysplasia, moderate dysplasia, severe dysplasia or carcinoma‐in‐situ.

People diagnosed with severe dysplasia and above were advised to undergo surgery or endoscopic therapy. For high‐grade dysplasia, carcinoma in situ and intramucosal carcinoma, local therapies were used, including endoscopic mucosal resection (EMR), endoscopic submucosal dissection (ESD), multiring banded mucosal excision (MBM), or radiofrequency ablation (RFA). For submucosal cancers (infiltration depth ≥ 200 μm) and advanced esophageal cancers, esophagectomy, radiotherapy, and other conventional treatments were used for treatment. For individuals diagnosed with mild or moderate dysplasia, endoscopic surveillance was recommended after 3–5 years or 1 year, respectively.

All individuals participated in the screening took a risk factor survey before endoscopy by trained investigators using a uniform questionnaire. In the first year of screening, 20% of the individuals in the unscreened group were invited to complete the same questionnaire.

### 2.3 Outcomes and follow‐up

The primary outcomes were the detection of esophageal cancer and its precancerous lesions within 1 year after baseline screening (screen‐detected cases), and the occurrence and death of esophageal cancer during follow‐up (follow‐up detected cases). We re‐classified precancerous lesions into low‐grade intraepithelial neoplasia (LGIN) and high‐grade intraepithelial neoplasia (HGIN) in accordance with diagnostic terms recommended by the International Agency for Research on Cancer.^16^ People diagnosed with HGIN and above at baseline were identified as positive cases. The diagnoses of esophageal cancer were classified according to the International Classification of Diseases (10th revision), and the codes included C15, C15.0, C15.1, C15.2, C15.3, C15.4, C15.5, C15.8 and C15.9.

The demographic information of the target population, including gender and date of birth, was derived from the household registration system. The information on cancer incidence and mortality were obtained by matching the target populations with the cancer registration and death surveillance database. All participants were followed up to 31 December 2015.

### 2.4 Statistical analyses

Multiple indicators related with outcomes were evaluated in the analysis. The participation rate, biopsy rate, and detection rate were described as proportions. The follow‐up time was defined from cohort entry date to the date of outcome onset, death or the end of follow‐up. For the screened individuals, the cohort entry date was the screening date, and for those who refused to screen, the invitation date was used as the cohort entry date. We used the Cox proportional hazards regression model to calculate the hazard ratios (HRs) and 95%CIs. The number needed to screen was calculated as the inverse of risk difference to measure the effect of screening strategy.^17^ A sensitivity analysis was performed to evaluate the effectiveness of screening based on a propensity‐matched cohort. Propensity scores were computed by using variables including age, sex, region and cohort entry year, and a nearest‐neighbor 1:1 matching scheme with a caliper size of 0.001 was used for matching.

Statistical analysis was completed using STATA V.14.0 (STATA, College Station, Texas, USA). The study was approved by the independent ethics committee of Cancer Hospital Chinese Academy of Medical Sciences (approval number: 16–171/1250).

## 3 RESULTS

The baseline characteristics of study population are summarized in Table 1. There were 113,340 participants in the screened group and 224,677 participants in the unscreened group. The median follow‐up times for screened group and unscreened group were 5.26 years and 5.65 years, respectively. Screening participants had a median age of 53 years, and 45.07% of the participants were male. A higher participation rate was observed in women (36.40%) than that in men (30.59%). The participation rate increased with age except for 65–69 years. Younger people (aged 40–44) and older people (aged 65–69) seemed to have lower participation rates, both less than 30%.

**TABLE 1:** Baseline characteristics of study population.

Characteristics	Screened (*N* = 113,340)	Not screened (*N* = 224,677)	Total (*N* = 338,017)	Participation rate (%)
Sex, *N* (%)				
Men	51,080 (45.07)	115,879 (51.58)	166,959 (49.39)	30.59
Women	62,260 (54.93)	108,798 (48.42)	171,058 (50.61)	36.40
Age at entry, *N* (%)				
40–44 y	22,659 (19.99)	58,910 (26.22)	81,569 (24.13)	27.78
45–49 y	22,977 (20.27)	45,037 (20.05)	68,014 (20.12)	33.78
50–54 y	21,506 (18.97)	38,599 (17.18)	60,105 (17.78)	35.78
55–59 y	22,250 (19.63)	36,653 (16.31)	58,903 (17.43)	37.77
60–64 y	15,873 (14.00)	25,671 (11.43)	41,544 (12.29)	38.21
65–69 y	8075 (7.12)	19,807 (8.82)	27,882 (8.25)	28.96
Area, *N* (%)				
Cxian	22,135 (19.53)	49,788 (22.16)	71,923 (21.28)	30.78
Feicheng	23,470 (20.71)	18,718 (8.33)	42,188 (12.48)	55.63
Linzhou	19,805 (17.47)	38,333 (17.06)	58,138 (17.20)	34.07
Yangcheng	12,460 (10.99)	41,081 (18.28)	53,541 (15.84)	23.27
Yanting	22,702 (20.03)	43,500 (19.36)	66,202 (19.59)	34.29
Yangzhong	12,768 (11.27)	33,257 (14.80)	46,025 (13.62)	27.74

The detection rates of positive cases diagnosed with HGIN and EC increased significantly with age (*p* < 0.001) (Table 2). They ranged from 0.37% and 0.15% in the age group of 40–44 years to 3.21% and 2.41% in the age group of 65–69 years in men and women, respectively. With the increase of age from 40–44 to 65–69 years, the diagnostic yield of positive case per 10,000 endoscopies increased correspondingly from 37 to 321 in men, and from 15 to 241 in women. After considering the impact of participation rate, the diagnostic yield of positive case per 10,000 invitations reached the peak in the 60–64 years old group, followed by 65–69 and 55–59 age group.

**TABLE 2:** The detection rate for esophageal cancer and precancerous lesions.

Age categories	Eligible invitees	Underwent endoscopy (%)	Took biopsies (%)	Detection rate, *n* (%)			Diagnostic yield of positive case^a^ per 10,000 endoscopies	Diagnostic yield of positive case^a^ per 10,000 invitations
				LGIN	HGIN	EC		
Men								
40–44	41,018	9768 (23.81)	5883 (60.23)	370 (3.79)	25 (0.26)	11 (0.11)	37	9
45–49	33,725	9966 (29.55)	5832 (58.52)	656 (6.58)	46 (0.46)	22 (0.22)	68	20
50–54	29,924	9485 (31.70)	6452 (68.02)	897 (9.46)	98 (1.03)	52 (0.55)	158	50
55–59	29,034	10,318 (35.54)	6790 (65.81)	1181 (11.45)	145 (1.41)	67 (0.65)	205	73
60–64	20,059	7615 (37.96)	5079 (66.70)	967 (12.70)	138 (1.81)	65 (0.85)	267	101
65–69	13,199	3928 (29.76)	2666 (67.87)	508 (12.93)	89 (2.27)	37 (0.94)	321	95
Women								
40–44	40,551	12,891 (31.79)	7037 (54.59)	344 (3.52)	15 (0.15)	4 (0.03)	15	5
45–49	34,289	13,011 (37.95)	6738 (51.79)	623 (6.25)	43 (0.43)	11 (0.08)	42	16
50–54	30,181	12,021 (39.83)	7505 (62.43)	951 (10.03)	83 (0.88)	26 (0.22)	91	36
55–59	29,869	11,932 (39.95)	7223 (60.53)	1214 (11.77)	144 (1.40)	39 (0.33)	153	61
60–64	21,485	8258 (38.44)	5082 (61.54)	929 (12.2)	125 (1.64)	50 (0.61)	212	81
65–69	14,683	4147 (28.24)	2446 (58.98)	444 (11.3)	76 (1.93)	24 (0.58)	241	68

Abbreviation: EC, esophageal cancer.

^a^ Patients diagnosed with HGIN and EC.

During screening and subsequent follow‐up, a total of 4023 people were diagnosed with esophageal cancer and 1857 of them died from it (Table 3). The majority of incident and death cases occurred after the age of 50, accounting for approximately 85% of all cases, and about half of the cases occurred after the age of 60. Overall, the incidence and mortality were reduced by 23% (HR =0.77, 95%CI:0.72–0.82, *p* < 0.001) and 48% (HR = 0.52, 95%CI:0.46–0.58, *p* < 0.001) in those undertaking screening. Compared with unscreened group, initiation of endoscopic screening after 55 years was associated with a reduced risk of incident esophageal cancer, and initiation of endoscopic screening after 45 years was associated with a reduced risk of death from esophageal cancer. The results of propensity score‐matched analysis were consistent with those of the primary analysis (Tables S1 and S2).

**TABLE 3:** Hazard ratio and number needed to screen among different age groups.

Age categories	Screened (*N* = 113,340)		Not screened (*N* = 224,677)		Hazard ratio (95%CI)	*p* value	NNS to prevent one event^a^
	Cases	Rate (95%CI)	Cases	Rate (95%CI)			
Incidence							
40–44	60	43.07 (33.44–55.47)	171	47.03 (40.48–54.63)	0.99 (0.73–1.34)	0.938	4703
45–49	121	92.90 (77.74–111.02)	270	99.67 (88.46–112.29)	0.94 (0.76–1.18)	0.605	2634
50–54	227	174.06 (152.83–198.24)	479	194.13 (177.50–212.31)	0.86 (0.73–1.01)	0.072	925
55–59	286	230.01 (204.84–258.27)	705	322.26 (299.33–346.95)	0.72 (0.63–0.83)	<0.001	214
60–64	282	331.01 (294.54–371.98)	653	435.89 (403.70–470.63)	0.77 (0.67–0.89)	<0.001	187
65–69	151	358.57 (305.70–420.57)	618	548.05 (506.50–593.01)	0.65 (0.55–0.79)	<0.001	104
Mortality							
40–44	21	15.05 (9.81–23.08)	81	22.25 (17.90–27.66)	0.74 (0.45–1.22)	0.243	2725
45–49	31	23.72 (16.68–33.73)	124	45.67 (38.30–54.45)	0.53 (0.35–0.79)	0.002	896
50–54	75	57.18 (45.60–71.70)	220	88.75 (77.77–101.29)	0.67 (0.51–0.87)	0.003	619
55–59	91	72.62 (59.13–89.18)	320	145.25 (130.18–162.07)	0.51 (0.40–0.65)	<0.001	272
60–64	98	113.67 (93.25–138.56)	361	238.86 (215.45–264.82)	0.50 (0.40–0.63)	<0.001	158
65–69	56	131.44 (101.15–170.80)	379	332.63 (300.77–367.86)	0.42 (0.32–0.56)	<0.001	98

^a^ NNS to prevent one event at 5 years follow‐up.

The NNSs to prevent one incident case of esophageal cancer decreased from 4703 in age group of 40–44 years to 104 in age group of 65–69 years, and the NNSs to prevent one death case from esophageal cancer decreased from 2725 in age group of 40–44 years to 98 in age groups of 65–69 years.

**FIGURE 1:**
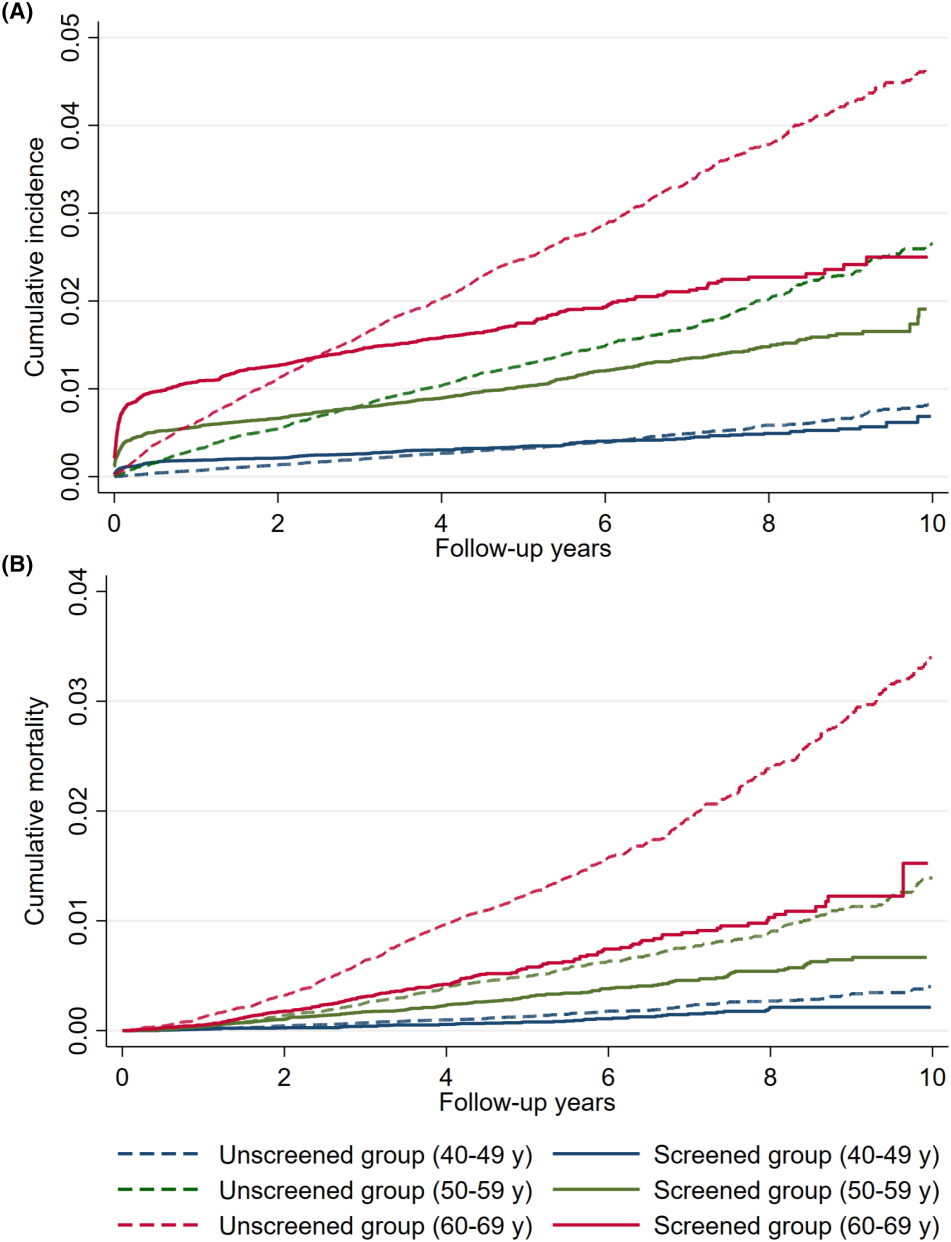
(A) Cumulative incidence and (B) Cumulative mortality in screened and unscreened groups by age at initiation of endoscopic screening.

The cumulative incidence and mortality curves between screened and unscreened group at different initiation age were shown in Figure 1 and Figures S1 and S2. Compared with unscreened group, initiation of screening after 50 years was associated with a greater reduction in cumulative incidence and mortality in 10 years follow‐up compared with initiation before 50 years. Table S3 showed the comparison of cumulative risk between screened group and unscreened group. For all ages, the screened group had lower 5‐year cumulative incidence and mortality rates than the unscreened group.

**FIGURE 2:**
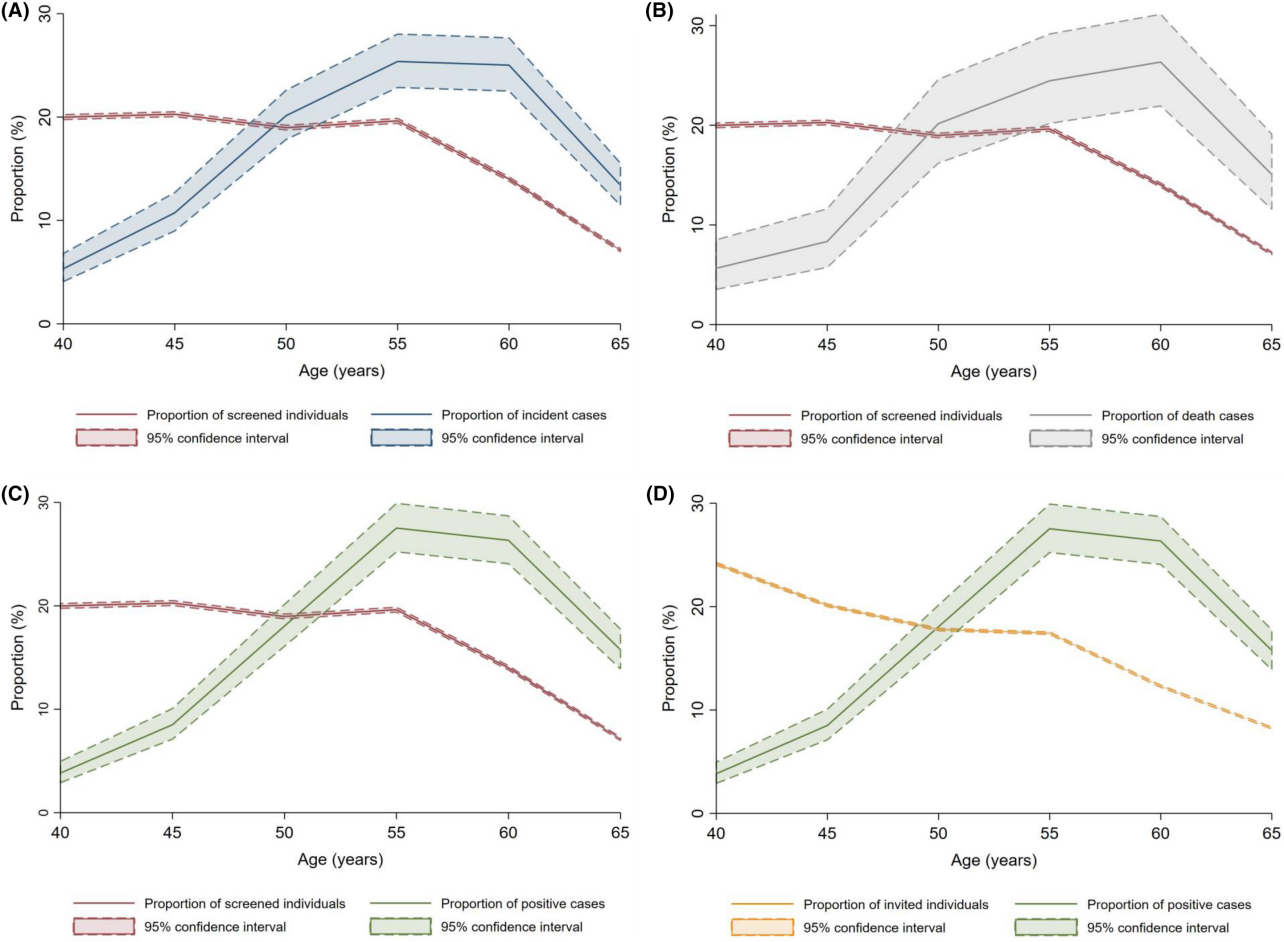
(A) Proportions of screened individuals and incident cases. (B) Proportions of screened individuals and death cases. (C) Proportions of screened individuals and positive cases. (D) Proportions of invited individuals and positive cases.

Figure 2 described the relationship between proportions of screened individuals and incident cases (Figure 2A), screened individuals and death cases (Figure 2B), screened individuals and positive cases (Figure 2C), as well as invited individuals and positive cases (Figure 2D) in each age group. The proportional curve of screened individuals crossed with proportional curve of incident cases around the age of 50 (Figure 2A). And the same trends were observed in other three comparisons (Figure 2B–D), indicating that the balance between screening burden and benefits is around 50 years of age, regardless of the outcomes.

We compared the number of screened individuals, missed positive cases, incident cases and deaths, as well as HR and NNS according to screening strategies at different starting ages (Table 4). When the screening age is increased to 50, it reduced the screening demand and NNS by 40% and 55%, and resulted in 12% of detectable positive cases, 16% of preventable incident cases and 14% of preventable deaths being missed. The hazard ratio was 0.73 (95%CI: 0.67–0.78) in term of incidence and 0.49 (95%CI: 0.43–0.55) in term of mortality.

**TABLE 4:** Comparisons of screening strategies with different starting ages.

Screening strategies age range	Screened group								Hazard ratio of incidence (95%CI)^a^	Hazard ratio of mortality (95%CI)^a^	NNS to prevent one case^b^ (95%CI)	NNS to prevent one death^b^ (95%CI)
	No. screened	Decreased demand (%)	Cases	Missed cases (%)	Deaths	Missed deaths (%)	Positive cases	Missed positive cases (%)				
40–69	113,340	—	1127	—	372	—	1435	—	0.77 (0.72–0.82)	0.52 (0.46–0.58)	488 (373–710)	381 (331–450)
45–69	90,681	22,659 (19.99)	1067	60 (5.32)	351	21 (5.65)	1380	55 (3.83)	0.75 (0.70–0.81)	0.50 (0.44–0.56)	301 (243–399)	275 (241–320)
50–69	67,704	45,636 (40.26)	946	181 (16.06)	320	52 (13.98)	1258	177 (12.33)	0.73 (0.67–0.78)	0.49 (0.43–0.55)	218 (178–282)	216 (189–252)
55–69	46,198	67,142 (59.24)	719	408 (36.20)	245	127 (34.14)	999	436 (30.38)	0.70 (0.64–0.76)	0.46 (0.40–0.53)	157 (130–202)	162 (142–190)
60–69	23,948	89,392 (78.87)	433	694 (61.58)	154	218 (58.60)	604	831 (57.91)	0.71 (0.63–0.79)	0.45 (0.38–0.54)	136 (107–191)	124 (106–150)
65–69	8075	105,265 (92.88)	151	976 (86.60)	56	316 (84.95)	226	1209 (84.25)	0.65 (0.55–0.79)	0.42 (0.32–0.56)	104 (77–169)	98 (80–131)

^a^ NNS to prevent one event at 5 years follow‐up.

^b^ Comparison between screened and unscreened group.

## 4 DISCUSSION

In this study, we comprehensively evaluated the impact of starting age for endoscopic screening of esophageal cancer by multiple outcomes based on a large‐sample multicenter cohort. The results indicated a favorable benefit‐to‐burden balance for postponing endoscopic screening to 50 years of age in high‐risk areas of esophageal cancer in China.

There are currently no clear and convincing evidence regarding the starting age of esophageal cancer screening. Previous studies showed inconsistent results and were limited due to small sample size and simplistic outcome evaluation. Feng et al found that screening is effective in reducing the mortality of esophageal cancer in 40–49 and 50–59 age group and recommended that people in high prevalence area of esophageal squamous cell carcinoma (ESCC) had one time endoscopy at their 50 years since the 50–59 age group had a lower NNS^13^. Wei et al suggested that the first screening could be postponed to 50 years based on the findings that individuals aged 50–69 years had 3.1 times higher cumulative incidence of ESCC than individuals aged 40–49 years.^14^ Yang et al^18^ conducted cost–benefit analyses to compare 12 endoscopic screening strategies, and found that screening once at age 50 years was the cheapest strategy but saves fewer life years, while Wu et al^19^ estimated the cost‐effectiveness of 36 strategies and suggested that endoscopic screening initiating at 40 years and repeated every 1–3 years is cost‐effective for the general population in high‐risk regions. The present study evaluated multiple outcomes to explore the optimal starting age for esophageal cancer screening based on a large cohort, which could provide strong evidence for the determination of starting age for esophageal cancer screening.

The benefits of improving screening age can be attribute to increased participant rate, detection rate, and screening effect. Participation rate is an important determinant of the effectiveness in population‐based screening. Our findings suggested that older people (50–69 years old) had a higher participation rate than younger people (40–49 years old), which were consistent with the results of previous studies.^5^, ^20^ The older people are more willing to participate in the screening since they are more concerned about their own health, and the exception for the 65–69 age group may due in part to the increased risk of invasive examination. The detection rate is mainly affected by the incidence level and the sensitivity of the detection method. With the rapid increase in the incidence of esophageal cancer after the age of 40, more positive cases can be detected early through endoscopy with biopsy, which is the gold standard for diagnosis of esophageal cancer, and lead to an increase in the detection rate. The screening effect reflects the magnitude of the reductions in incidence and mortality and is also related with age. No significant reduction was noted in incidence of esophageal cancer in individuals aged 40–49 and in mortality of esophageal cancer in individuals aged 40–44, suggesting the limited effect of screening on young people. In conclusion, increasing the screening age to 50 years can achieve higher participation rate, detection rate and screening effect, which will reduce the burden of screening, improve the efficiency, and avoid overscreening.

The risk of increasing the screening age is the reduced number of individuals who are prevented from esophageal cancer through early detection and early treatment. Decision makers should consider the tradeoff between benefit and risk based on the local health resources and social‐economic level in determination of the target age for screening. Screening at the age of 40 instead of 50 gains more benefits by detecting and preventing more cases. However, in areas with limited resources, we recommend that the starting age of screening could be defined as 50 years based on the following reasons: (1) Starting screening at the age of 50 instead of 40 can reduce a great number of demands for screening, while the loss of benefit is relatively small. When the screening age is increased to 50, the demand for screening can be reduced by 40%, with the sacrifice of 12% of detectable positive cases, 16% of preventable incident cases and 14% of preventable deaths. (2) A huge gap was observed in NNS between individuals who were in their 40 s and 50 s. The NNS to prevent one incident case esophageal cancer is 4703 and 2634 in individuals aged 40–44 and 45–49, respectively, while it is 925 and 214 in individuals aged 50–54 and 55–59, respectively. (3) Screening has limited effect on people aged 40–49 but could cause additional risk such as complications. Increasing the starting age to 50 can avoid the unnecessary endoscopic examinations and improve the effectiveness of screening.

The existing guidelines for population‐based endoscopic screening are for gastric cancer and their recommendation on starting age are different. In Japan, the latest guideline for gastric cancer screening suggests that individuals start a biennial or triennial endoscopic screening at the age of 50,^21^ while in South Korea, the national cancer screening program for gastric cancer invites individual aged 40 years or above to undergo endoscopy or an upper gastrointestinal series every 2 years.^22^ Unlike developed countries that have the ability to implement endoscopic screening in the general population, high incidence areas of esophageal cancer usually have low‐ and middle‐income and insufficient health resources, and the endoscopy capacity can only cover a limited population. Therefore, based on the recent decrease in incidence of esophageal cancer in China, increasing the starting age of endoscopic screening can concentrate the target population for an effective screening and achieve a favorable benefit‐to‐burden balance.

The screening program evaluated in this study consisted of one‐time screening at baseline and endoscopic surveillance for positive cases. Repeated screening of individuals diagnosed with mild or moderate dysplasia could help monitor disease progression, guide early diagnosis and treatment, and further reduced the incidence and mortality of esophageal cancer. A total of 5180 individuals took at least one endoscopic follow‐up during the study period, accounting for 4.6% of screened individuals, which contributed to the reduction on incidence and mortality. Health economic studies^19^, ^23^ showed that compared with no screening, both one‐time screening and repeated screening decreased are cost‐effective and repeated screening every 2 years would be the optimal strategy.^23^ However, for high prevalence areas with limited health resources, at least one screening with repeated screening for positive cases is recommended if there is no capacity for repeated screening.

In addition, the estimation of the follow‐up time and the method of follow‐up may also have an impact on the results. Since we used the invitation date as the cohort entry date for the unscreened group, which was usually earlier than the date of screening, the median follow‐up time was longer for the unscreened group than for the screened group. This would overestimate the follow‐up duration for the unscreened group, resulting in low calculated incidence and mortality rates, and would overestimate the effectiveness of screening. We conducted passive follow‐up based on the cancer registry system, so that the incidence and mortality outcomes of all screened and unscreened individuals could be tracked. However, there is often a significant delay and more missed diagnosis for passive matching to cancer registry compared to active follow‐up to ascertain the outcomes. Therefore, the lack of active follow‐up in the unscreened group might lead to missed diagnoses, resulting in overestimation of screening effectiveness.

To our knowledge, this is the first study to evaluate the impact of starting age of endoscopic screening for esophageal cancer based on data from a large sample multicenter cohort. The strength of this study is the comprehensive evaluation of multiple outcomes, including participation rate, detection rate, incidence, mortality, cumulative incidence and mortality, as well as estimated indicators such as diagnostic yield, HR and NNS. Compared to modeling studies, the present results provide real‐world evidence that can be used directly as well as important data that can be used for subsequent simulation‐based cost‐effectiveness analyses.

There are several limitations in this study. First, this study is based on real‐world data, and there is inevitable selection bias and information bias. Due to the lack of risk factors for all unscreened individuals, we could only compare the risk factors between screened and unscreened groups in a limited sample (Table S4). The results showed that participants in the screened group had more frequently reported of risk factors of esophageal cancer, including poor drinking water, lower household income and a family history of cancer. This may be due to the health education conducted during enrollment, encouraging those with risk factors to participate in screening. In addition, the screening was free and those with low income would be more likely to participate. We also performed sensitivity analysis based on propensity score matching cohort using existing variables and the results showed similar trend to the original analysis. Second, most of the cases in this study were esophageal squamous cell carcinoma, resulting to the limited evidence for screening for adenocarcinoma. Finally, we use the screening number of participants to reflect the screening burden, without considering the direct and indirect costs of screening and related treatments. Since economic status plays an important role in the determination of starting age of screening, cost–benefit analysis and cost‐effectiveness analysis are required to verify the results in the further study.

In conclusion, this study provides evidence‐based recommendations on starting age of esophageal cancer screening in high‐risk areas. The findings could be used to guide the individuals to participate in screening, and help to improve the efficiency of screening and avoid overscreening.

## AUTHOR CONTRIBUTIONS

**Ru Chen:** Conceptualization (lead); formal analysis (lead); writing – original draft (lead); writing – review and editing (lead). **Lizhou Dou:** Conceptualization (equal); writing – original draft (equal). **Jiachen Zhou:** Formal analysis (supporting). **Guohui Song:** Data curation (equal). **Bianyun Li:** Data curation (equal). **Deli Zhao:** Data curation (equal). **Zhaolai Hua:** Data curation (equal). **Xinzheng Wang:** Data curation (equal). **Jun Li:** Data curation (equal). **Changqing Hao:** Data curation (supporting). **Yanyan Li:** Data curation (equal). **Xiang Feng:** Data curation (equal). **Lin Li:** Data curation (equal). **Wenqiang Wei:** Conceptualization (lead); supervision (lead); writing – original draft (lead); writing – review and editing (lead). **Guiqi Wang:** Conceptualization (lead); supervision (lead); writing – original draft (supporting); writing – review and editing (supporting).

## FUNDING INFORMATION

This study is supported by National Natural Science Foundation of China (81903403, 81974493), Beijing Natural Science Foundation (7204294), National Science & Technology Fundamental Resources Investigation Program of China (2019FY101101), and the Chinese Academic of Medical Sciences Innovation Fund for Medical Sciences (2021‐I2M‐1‐013).

## CONFLICT OF INTEREST STATEMENT

The authors disclose no conflicts.

## Supporting information


Appendix S1

## Data Availability

The data supporting the findings of this study are available from the corresponding authors upon reasonable request.
