# Supplementary material for: Optimal starting age of endoscopic screening for esophageal cancer in China: A multicenter prospective cohort study
Source: Cancer Med. 2023 Apr 7;12(8):9988–98. doi: 10.1002/cam4.5727 (PMC10166951; doi:10.1002/cam4.5727)

**Supplementary materials**

**Optimal starting age of endoscopic screening for esophageal cancer in China: a multicenter prospective cohort study**

Table S1 Baseline characteristics of screened and not screened subjects before and after matching on propensity score

Table S2 Hazard ratio and number needed to screen among different age groups based on propensity score matched cohort

Table S3 Comparison of potential risk factors between the screened and unscreened group

Figure S1 Cumulative incidence in screened and unscreened groups by age at initiation of endoscopic screening

Figure S2 Cumulative mortality in screened and unscreened groups by age at initiation of endoscopic screening

Figure S3 Cumulative risk of screened and unscreened groups by age

**Table S1 Baseline characteristics of screened and not screened subjects before and after matching on propensity score**

| **Baseline characteristics** | **Unmatched** | | | **Propensity score-matched** | | |
| --- | --- | --- | --- | --- | --- | --- |
|  | **Screened group** | **Not screened group** | **P value** | **Screened group** | **Not screened group** | **P value** |
| Number | 113340 | 224677 |  | 96300 | 96300 |  |
| Area, N(%) |  |  | <0.001 |  |  | 1.000 |
| Cixian | 22135(19.53) | 49788(22.16) |  | 21657(22.49) | 21657(22.49) |  |
| Feicheng | 23470(20.71) | 18718(8.33) |  | 16787(17.43) | 16787(17.43) |  |
| Linzhou | 19805(17.47) | 38333(17.06) |  | 14965(15.54) | 14965(15.54) |  |
| Yangcheng | 12460(10.99) | 41081(18.28) |  | 12105(12.57) | 12105(12.57) |  |
| Yanting | 22702(20.03) | 43500(19.36) |  | 18902(19.63) | 18902(19.63) |  |
| Yangzhong | 12768(11.27) | 33257(14.80) |  | 11884(12.34) | 11884(12.34) |  |
| Sex, N(%) |  |  | <0.001 |  |  | 0.816 |
| Men | 51080(45.07) | 115879(51.58) |  | 45446(47.19) | 45395(47.14) |  |
| Women | 62260(54.93) | 108798(48.42) |  | 50854(52.81) | 50905(52.86) |  |
| Age at entry, N(%) |  |  | <0.001 |  |  | 0.964 |
| 40-44 yr | 22659(19.99) | 58910(26.22) |  | 20125(20.9) | 20074(20.85) |  |
| 45-49 yr | 22977(20.27) | 45037(20.05) |  | 19644(20.4) | 19644(20.4) |  |
| 50-54 yr | 21506(18.97) | 38599(17.18) |  | 17252(17.91) | 17395(18.06) |  |
| 55-59 yr | 22250(19.63) | 36653(16.31) |  | 18520(19.23) | 18520(19.23) |  |
| 60-64 yr | 15873(14) | 25671(11.43) |  | 13185(13.69) | 13093(13.6) |  |
| 65-69 yr | 8075(7.12) | 19807(8.82) |  | 7574(7.87) | 7574(7.87) |  |
| Cohort entry year, N(%) |  |  | <0.001 |  |  | 1.000 |
| 2005 | 3210(2.83) | 16671(7.42) |  | 3145(3.27) | 3145(3.27) |  |
| 2006 | 8939(7.89) | 10901(4.85) |  | 5256(5.46) | 5205(5.4) |  |
| 2007 | 13532(11.94) | 12410(5.52) |  | 9142(9.49) | 9142(9.49) |  |
| 2008 | 8194(7.23) | 7163(3.19) |  | 5846(6.07) | 5897(6.12) |  |
| 2009 | 9863(8.70) | 12585(5.60) |  | 7652(7.95) | 7652(7.95) |  |
| 2010 | 21923(19.34) | 59205(26.35) |  | 21029(21.84) | 21029(21.84) |  |
| 2011 | 25268(22.29) | 62663(27.89) |  | 23226(24.12) | 23226(24.12) |  |
| 2012 | 22411(19.77) | 43079(19.17) |  | 21004(21.81) | 21004(21.81) |  |

**Table S2 Hazard ratio and number needed to screen among different age groups based on propensity score matched cohort**

| **Age categories** | **Screened (N=96300)** | | **Not screened(N=96300)** | | **Hazard ratio (95%CI)** | **P value** | **NNS to prevent one event** ^a^ |
| --- | --- | --- | --- | --- | --- | --- | --- |
|  | **Cases** | **Rate (95%CI)** | **Cases** | **Rate (95%CI)** |  |  |  |
| Incidence |  |  |  |  |  |  |  |
| 40-44 | 50 | 41.82(31.7-55.18) | 75 | 57.4(45.77-71.97) | 0.72(0.51-1.04) | 0.077 | 1249 |
| 45-49 | 101 | 93.73(77.12-113.91) | 124 | 104.6(87.72-124.73) | 0.88(0.68-1.15) | 0.360 | 1706 |
| 50-54 | 181 | 180.79(156.28-209.14) | 249 | 227.21(200.67-257.26) | 0.78(0.64-0.94) | 0.010 | 416 |
| 55-59 | 238 | 239.67(211.08-272.14) | 340 | 315.89(284.04-351.32) | 0.74(0.63-0.87) | 0.001 | 258 |
| 60-64 | 243 | 356.23(314.15-403.96) | 316 | 431.07(386.07-481.31) | 0.80(0.68-0.95) | 0.009 | 261 |
| 65-69 | 140 | 363.9(308.35-429.46) | 227 | 558.56(490.43-636.16) | 0.63(0.51-0.78) | <0.001 | 103 |
| Mortality |  |  |  |  |  |  |  |
| 40-44 | 19 | 15.87(10.12-24.88) | 35 | 26.74(19.20-37.25) | 0.65(0.37-1.15) | 0.139 | 1805 |
| 45-49 | 27 | 24.98(17.13-36.42) | 63 | 53.01(41.41-67.86) | 0.49(0.31-0.77) | 0.002 | 703 |
| 50-54 | 57 | 56.59(43.65-73.37) | 112 | 101.63(84.45-122.31) | 0.57(0.42-0.79) | 0.001 | 439 |
| 55-59 | 77 | 76.95(61.55-96.21) | 158 | 145.79(124.74-170.39) | 0.53(0.40-0.70) | <0.001 | 288 |
| 60-64 | 83 | 120.19(96.92-149.04) | 181 | 244.75(211.57-283.14) | 0.50(0.38-0.64) | <0.001 | 159 |
| 65-69 | 47 | 120.76(90.73-160.72) | 138 | 336.13(284.47-397.15) | 0.36(0.26-0.50) | <0.001 | 93 |
| ^a^ NNS to prevent one event at 5 years follow-up | | | | | | | |

**Table S3 Comparison of potential risk factors between the screened and unscreened group**

| **Characteristic** | **Screened group (N=3179) ^a^** | | **Unscreened group (N=3280) ^a^** | | **P value** |
| --- | --- | --- | --- | --- | --- |
|  | **No** | **%** | **No** | **%** |  |
| Sex |  |  |  |  | 0.002 |
| Male | 1415 | 44.51 | 1586 | 48.35 |  |
| Female | 1764 | 55.49 | 1694 | 51.65 |  |
| Age at entry |  |  |  |  | <0.001 |
| 40-49 yr | 1380 | 42.07 | 1512 | 47.56 |  |
| 50-59 yr | 1158 | 35.30 | 1283 | 40.36 |  |
| 60-69 yr | 742 | 22.62 | 384 | 12.08 |  |
| Marriage |  |  |  |  | <0.001 |
| Married | 2984 | 93.87 | 2968 | 90.49 |  |
| Single/divorced/widowed | 195 | 6.13 | 312 | 9.51 |  |
| Household members, No. |  |  |  |  | <0.001 |
| <5 | 1847 | 58.17 | 2123 | 64.75 |  |
| >=5 | 1328 | 41.83 | 1156 | 35.25 |  |
| Household income |  |  |  |  | <0.001 |
| <=¥5000 | 1773 | 55.84 | 1433 | 43.97 |  |
| >¥5000 | 1402 | 44.16 | 1826 | 56.03 |  |
| Education |  |  |  |  | 0.293 |
| No primary education | 478 | 14.57 | 493 | 15.51 |  |
| Primary education or more | 2802 | 85.43 | 2686 | 84.49 |  |
| Drinking water |  |  |  |  | <0.001 |
| Shallow well or lake water | 1719 | 54.24 | 520 | 15.87 |  |
| Deep well or piped water | 1450 | 45.76 | 2756 | 84.13 |  |
| Smoking |  |  |  |  | 0.440 |
| No | 2354 | 74.05 | 2401 | 73.2 |  |
| Yes | 825 | 25.95 | 879 | 26.8 |  |
| Drinking alcohol |  |  |  |  | 0.626 |
| No | 2757 | 86.73 | 2858 | 87.13 |  |
| Yes | 422 | 13.27 | 422 | 12.87 |  |
| Diseases of digestive system |  | |  |  | 0.914 |
| No | 2737 | 86.1 | 2827 | 86.19 |  |
| Yes | 442 | 13.9 | 453 | 13.81 |  |
| Family history of cancer |  |  |  |  | <0.001 |
| No | 1974 | 62.09 | 2509 | 76.49 |  |
| Yes | 1205 | 37.91 | 771 | 23.51 |  |
| ^a^ Individuals interviewed in 2005 | | | | | |

**Figure S1 Cumulative incidence in screened and unscreened groups by age at initiation of endoscopic screening**


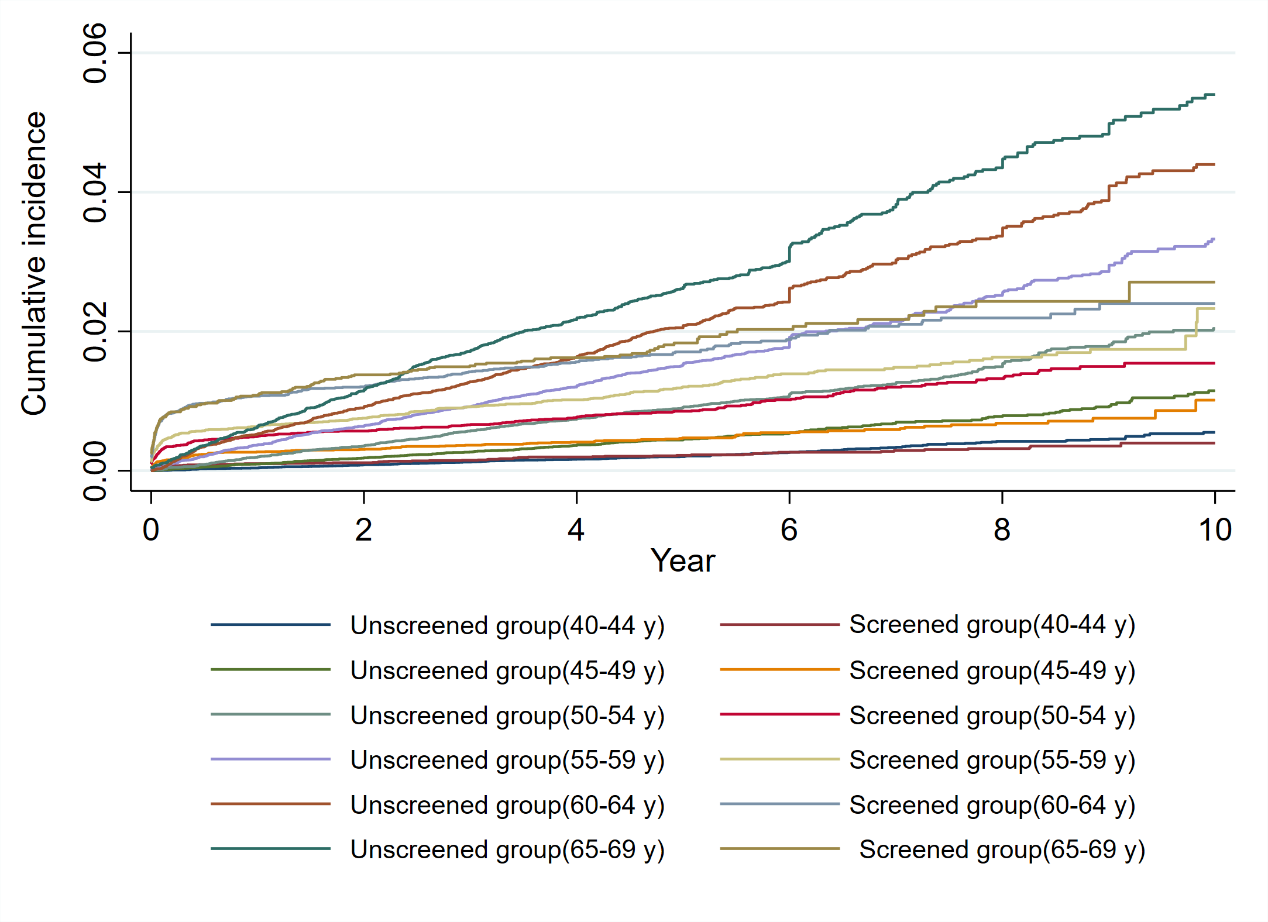


**Figure S2 Cumulative mortality in screened and unscreened groups by age at initiation of endoscopic screening**


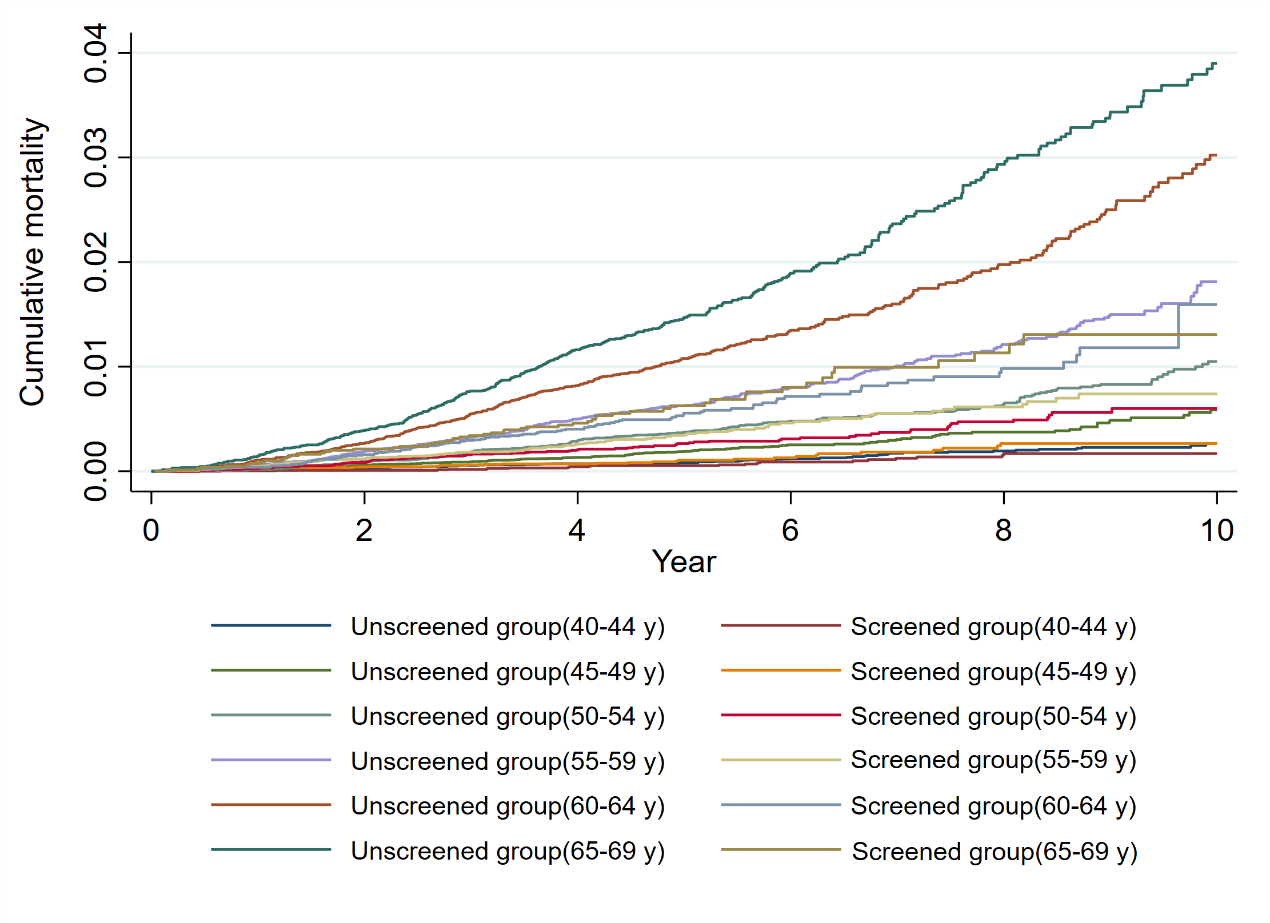


**Figure S3 Cumulative risk of screened and unscreened groups by age**


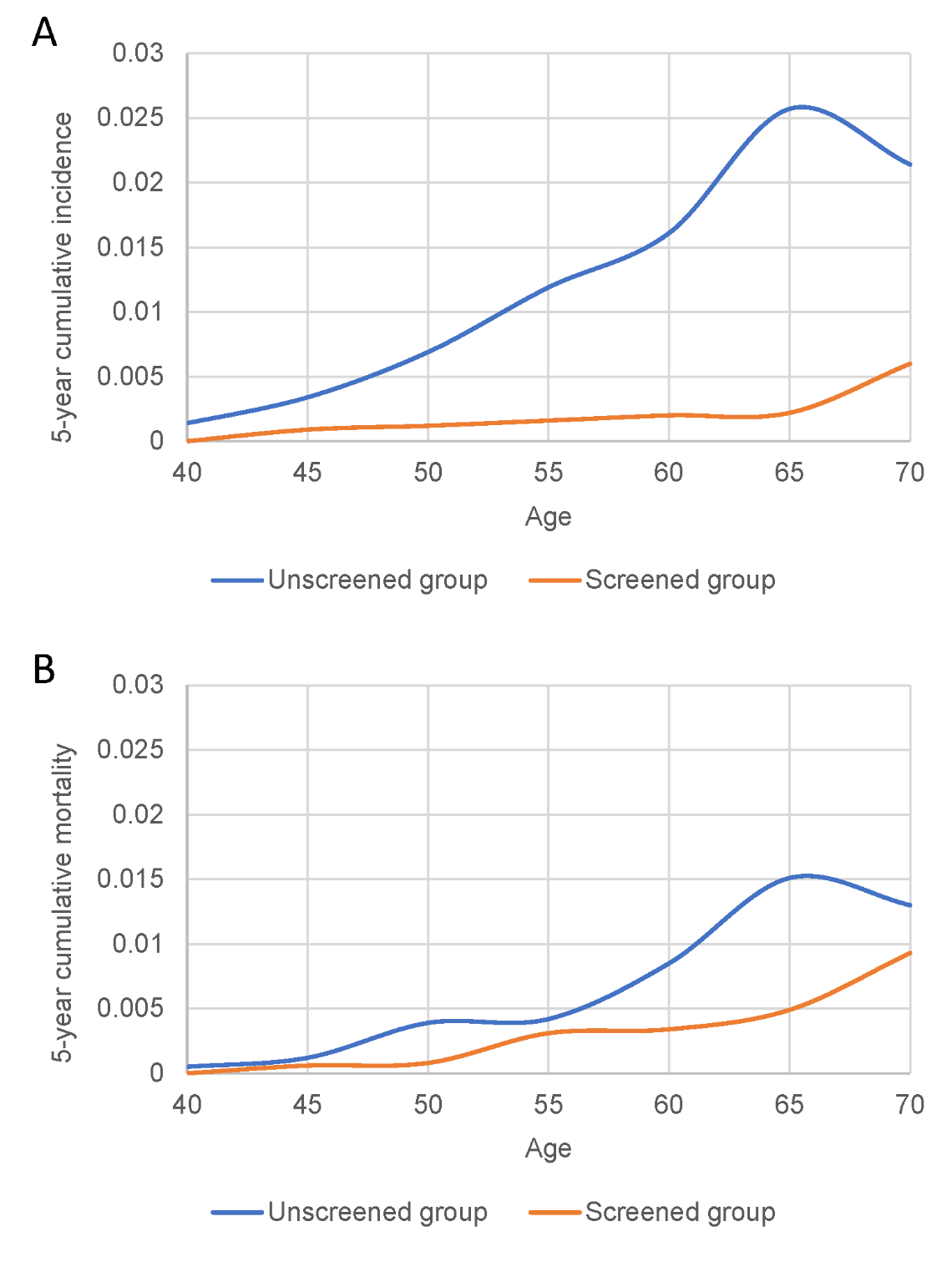

Supplement: Supplementary file 1 — Appendix S1 [file CAM4-12-9988-s001.docx]
